# Valuable Features in Mobile Health Apps for Patients and Consumers: Content Analysis of Apps and User Ratings

**DOI:** 10.2196/mhealth.4283

**Published:** 2015-05-13

**Authors:** Martin F Mendiola, Miriam Kalnicki, Sarah Lindenauer

**Affiliations:** ^1^SocialWellth, IncLas Vegas, NVUnited States

**Keywords:** mHealth, mobile apps, consumer preference, Affordable Care Act

## Abstract

**Background:**

The explosion of mobile phones with app capabilities coupled with increased expectations of the patient-consumers’ role in managing their care presents a unique opportunity to use mobile health (mHealth) apps.

**Objectives:**

The aim of this paper is to identify the features and characteristics most-valued by patient-consumers (“users”) that contribute positively to the rating of an app.

**Methods:**

A collection of 234 apps associated with reputable health organizations found in the medical, health, and fitness categories of the Apple iTunes store and Google Play marketplace was assessed manually for the presence of 12 app features and characteristics. Regression analysis was used to determine which, if any, contributed positively to a user’s rating of the app.

**Results:**

Analysis of these 12 features explained 9.3% (*R* ^2^=.093 n=234, *P*<.001) of the variation in an app’s rating, with only 5 reaching statistical significance. Of the 5 reaching statistical significance, plan or orders, export of data, usability, and cost contributed positively to a user’s rating, while the tracker feature detracted from it.

**Conclusions:**

These findings suggest that users appreciate features that save time over current methods and identify an app as valuable when it is simple and intuitive to use, provides specific instructions to better manage a condition, and shares data with designated individuals. Although tracking is a core function of most health apps, this feature may detract from a user’s experience when not executed properly. Further investigation into mHealth app features is worthwhile given the inability of the most common features to explain a large portion of an app’s rating. In the future, studies should focus on one category in the app store, specific diseases, or desired behavior change, and methods should include measuring the quality of each feature, both through manual assessment and evaluation of user reviews. Additional investigations into understanding the impact of synergistic features, incentives, social media, and gamification are also warranted to identify possible future trends.

## Introduction

The impact of recent health reform efforts are far-reaching, with perhaps one of the biggest shifts occurring in the convergence of clinical care delivery and consumer health. The Patient Protection and Affordable Care Act (PPACA) is a health care reform measure enacted in 2010 by the US Congress under President Barack Obama that seeks to make access to health care more affordable, efficient, and comprehensive for Americans. New mandates and incentives within PPACA call for a larger role for patients in health care, presenting an opportunity to incorporate and integrate digital health (wireless sensors, social networking, and mobile connectivity) into what Americans consider institutional health care (hospitals, physicians, and insurance plans). This has the potential to fundamentally change how patients manage their own health. PPACA was intended to be the catalyst, and technology the facilitator, that empowered patients to be more prominent participants in their own health management.

Expectations of increased patient enablement through the use of technology seem much more attainable since the introduction and proliferation of the mobile phone. Today, 90% of American adults own a cellular phone, and more than 70% own a mobile phone with app capabilities [1]. Of those with mobile phones, approximately 20% already use a mobile health (mHealth) app, and by the end of 2015 that number is expected to rise to 33% [2]. Although promising, mobile phones continue to present an untapped opportunity to reach more patient consumers (“users”), especially since 70% of adults track some sort of health indicator for themselves or a loved one through paper logs or other means [3].

The explosion of mobile phone ownership coupled with increased expectations of the users’ role in managing their care presents a unique opportunity for mHealth apps. Chronic diseases account for 75% of the $2.7 trillion in annual US health care spending [4], therefore tools like apps to help prevent, slow the progression of, or manage chronic disease are seen as valuable in helping to lower health care costs. Currently, identifying which health apps would be most effective for a population seems daunting, even more so for a specific individual [5]. Few research efforts have focused on understanding what a user values in a health app, a timely question considering that there are over 100,000 apps in the medical, health, and fitness sections of the Apple iTunes store and Google Play marketplace [6]. Additionally, the sustained use of any one app is low, where 68% of mobile phone users open ≤5 apps at least once a week [7] while 80%-90% percent of apps are used just once and then later deleted.

Early research in the digital health industry focused on identifying and listing mobile app features and characteristics for easy cataloguing [8,9], however, if users are expected to use mHealth apps regularly in order to play a larger role in their own care, then it is essential to gain a better understanding of the qualities and approaches that lead to long-term app usage. Arnhold et al examined the usability and functionality of a subset of mHealth apps specifically designed for diabetics [10]. They evaluated the impact of different app functions on user experience, and showed that although there are many apps that address the diabetes condition, very few offer more than just one or two basic functions. Those that offered more functions fared considerably worse when assessed for usability.

In order to promote sustained usage and positively influence health outcomes, it is critical that mHealth apps have a basis in behavioral science. Several studies have evaluated mHealth apps using frameworks rooted in behavior theory, with many focusing on identifying features that facilitate one of the following three basic psychological needs of the Self-Determination Theory (SDT) (1) autonomy, (2) competence, and (3) relatedness [11]. Autonomy refers to individuals’ desires to regulate behavior based on their own values and interests. In order for an mHealth app to encourage autonomous motivation, it must accurately portray the value of the associated behavior change, give users a choice in their interaction with the app, acknowledge users’ perspectives, and provide an action plan to support individuals and their needs. Competence refers to an individual’s need to feel capable and confident to change his/her behavior, and can be facilitated by providing relevant information, tools, resources, and feedback throughout the health journey. Relatedness refers to the degree of connectedness that an individual feels with others, and it can be fostered through an mHealth app by creating virtual communities, connecting to social media sites, or actively attempting to better understand the individual user.

A recent study by Choi et al [12] evaluated smoking cessation apps for both content and functionality using tenets from SDT. Findings revealed that most of the smoking cessation apps (94.3%) had at least one feature that employed one of the three basic SDT needs, but few (10.3%) addressed all three. While not specific to one theory, Dahlke et al recently investigated the number of health behavior and communication constructs applied in mobile phone cancer survivorship apps, and found that while some of the apps utilized theoretical elements of behavior change, there remains an overall need for more theory-based apps in the mHealth space [13]. However, neither of these studies examined the relative influence of the various features on user satisfaction or sustained use.

Whereas most digital health studies, including those mentioned above, have focused on apps targeting just one condition, Payne et al systematically reviewed 24 studies that utilized a variety of mHealth apps across a range of health behavior interventions [14]. These apps were examined to identify features and functions central to behavior change, and the findings suggested that all of the apps included some element that addressed a behavior change theory or strategy, although their relative influence was not reported.

The foundation for digital health studies has been built on cataloguing the number and types of features in apps addressing a particular disease state, determining the theoretical impact of certain features on user experience, and identifying the behavioral theories expressed in a subset of mHealth apps. Additional studies should explore specific app features across a breadth of wellness and medical apps that lead to a positive user experience and ultimately long-term behavior change. The features investigated should closely align with SDT and common usability principles, since these are two prominent components of long-term engagement. Through analysis of a subset of mHealth apps, this paper aimed to identify those features that are aligned with SDT and common usability principles, and are most-valued and have contributed positively to a user’s rating of the app in order to ultimately provide a roadmap for future mHealth app development.

## Methods

### General App Inclusion Criteria

First, a set of inclusion criteria was established to limit the scope of apps being evaluated prior to attempting to identify app features deemed valuable to a user. To create a group of comparable apps from the more than 100,000 mHealth apps in the Apple iTunes store and Google Play marketplace, the dataset was limited to apps that were associated with reputable health organizations: these were defined as developers or evaluators with relationships with content-credible health care entities (Textbox 1).

**Definitions of reputable health organization inclusion criteria.**

Requirement and description

- Clinical trial/research study
  - Have undergone a research study or clinical trial and had results published
- Food and Drug Administration (FDA)
  - Have been approved by the US FDA
- Government-approved
  - Have been developed or endorsed by a non-FDA government agency (eg, US Department of Veterans Affairs and Centers for Disease Control and Prevention)
- US hospital system-approved
  - Have been developed or endorsed by a US hospital system (eg, Cleveland Clinic and Carolinas Health System)
- US academic medical institution-approved
  - Have been developed or endorsed by a US academic medical institution (eg, Harvard Medical School and Vanderbilt University)
- Medical specialty society-approved
  - Have been developed or endorsed by a medical specialty society (eg, American College of Cardiology and American Society of Clinical Oncology)
- Non-profit health care organization-approved
  - Have been developed or endorsed by a non-profit health care organization (eg, American Diabetes Association and National Breast Cancer Foundation)
- Consumer organization with health focus-approved
  - Have been developed or endorsed by a national consumer company focused on health (eg, WebMD and Walgreens)
- US physician-approved
  - Have been developed or endorsed by a board-certified US physician
- Third-party payer-approved
  - Have been developed or endorsed by a private third-party insurance payer (eg, Aetna)
- Pharmaceutical or medical technology company-approved
  - Have been developed by a pharmaceutical or medical technology company (eg, Novartis Consumer Health and Medtronic)

Between March 19 and April 8, 2014, a list of the apps that met the inclusion criteria was compiled using information available from several systems. Using the PubMed and mHealth Evidence websites [15,16], the terms “iPhone,” “Android,” “Apple,” and “Google Play” were used separately as search queries to identify apps that had undergone a clinical trial or research study with published results. A relational database was created using the services of 42matters, a privately-held technology company that provides services for app discovery and analytics. This database contained the names, developers, and descriptions of the 100,000 mHealth apps in the Apple iTunes store and Google Play marketplace, and was used to identify any apps that referenced the name of any federal government agency, US hospital system, US academic medical institution, medical specialty society, private third-party insurance payer, pharmaceutical company, or medical technology company, as well as any notable non-profit health care organizations, national consumer companies focused on health, and board-certified US physicians that have a strong presence in mHealth. The apps that satisfied the reputable health organization criteria were then subjected to additional criteria related to purpose and functionality (Textbox 2).

**Additional purpose and functionality criteria.**

Criteria

- Individual user ratings (>25) exist for the app in the respective app store

- Health consumers are its primary audience, as determined through active use and exploration of the app. Some apps, particularly in the “Medical” category of the app stores, target health care professionals and students as a means of reference or supplementary training and were omitted from analysis

- Created for US audiences, sold in US app stores, and contain an English-language user interface (UI)
- Ability to function independently of a medical device
- No required special passwords or access codes associated with a provider or payer program (since access to these apps require the user to be a patient of a particular provider or member of a payer program in order to obtain an access code, and without it, the app’s features and functions could not be assessed)

Since the dataset used for analysis was pulled from two sources (Apple iTunes store and Google Play marketplace, duplicate apps (ie, identical features in the same app listed independently in both app stores) were eliminated. However, apps by the same developer, similarly named, but not having identical feature sets were treated as two different apps. Furthermore, the ratings were adjusted (Y2) to reflect a consistency in the presentation of the ratings, (ie, Google Play has continuous values to one tenth of a point, whereas Apple iTunes rounds ratings to the nearest half point). The app store ratings were converted into Bayesian Ratings (Y3) since there is growing support for the use of Bayesian analysis to assess any user-generated content, such as ratings, games, and cases that take into account individual judgment [17]. Lastly, with a recent spotlight shone on questionable techniques for increasing an app’s number of ratings [18], any apps that received an unusually large number of ratings (ie, >3000 individual user ratings) were deemed outliers and eliminated from consideration.

### Analysis of App Features and Characteristics

The remaining apps were downloaded and manually assessed for the presence of certain features or characteristics that have been studied in other published research [14] as a means to engage and change behavior (Table 1).

Using a binary system, apps were assigned a “1” to indicate the presence of a particular feature, or a “0” to indicate the absence. Only one attribute, cost, was assessed on 3 parameters because of the mutually exclusive cost options of free, free with in-app purchases, and paid, which were assigned values of “0,” “0.5,” and “1,” respectively. Usability, being a more subjective and complex characteristic, required additional analysis before being assigned a score. Each app was downloaded and functionality explored before being rated against five of Jakob Nielsen’s general principles for interaction design (Textbox 3) [19]. Apps that met a majority of the usability principles received a score of “1,” otherwise a “0” was assigned.

Regression analysis was performed using Microsoft Excel to investigate the features that influenced an individual user’s rating of an app. Since users are most often asked to rate an app only after they have begun using it regularly, ratings found in the app stores can serve as a proxy for assessing an app’s value to its users. In the end, all regressions were executed against the following 3 separate dependent (Y) variables (1) Y1: ratings, (2) Y2: adjusted ratings, and (3) Y3: Bayesian ratings.

The inclusion criteria variables were also evaluated as independent variables to confirm that they were not confounders and could be eliminated from consideration for further analysis. At this point, multiple regression analysis could not be performed because the number of variables under consideration exceeded the maximum capacity of Microsoft Excel. Therefore, simple linear regression analysis was performed with each independent variable against Y1, Y2, and Y3 to gauge if any app feature or inclusion criterion independently influenced the dependent variables.

Using the independent variables that exhibited at least minimal influence, multiple regression analysis was performed against the same dependent variables to determine whether a combination of features could explain a user’s rating of an app. Using a 95% confidence level, independent variables were eliminated based on *P* values, and the model was assessed for accuracy based on *F* statistic (primarily) and R-squared values. Correlation analysis was conducted to assess whether there were any pairwise associations between variables. Finally, the user reviews of a random sample of apps (10.3% of the total dataset (n=24) were assessed to determine whether users focused on the app features and characteristics addressed in the study.

**Table 1:** App features and characteristics.

App feature or characteristic	Description of feature or characteristic	Relevant construct(s) & principle(s)
Export of Data	Feature that allows the user to communicate or send information/ data to a health care provider (eg, email and EHR/PHR)	Relatedness (care team collaboration and support)
Gamification	Feature that offers points, badges, or movement through levels as a health objective is achieved or the more a patient is engaged (see Figure 1)	Autonomy (extrinsic motivation, engagement)
General education	Feature that provides basic educational material about a disease/condition, including causes, treatment, or management	Autonomy (intrinsic motivation); competence (knowledge)
Plan or orders	Feature that provides a plan of action for reaching target goal, including specific, executable steps to guide the process (see Figure 2)	Competence [actionable insights]; Autonomy [goal-setting]
Reminder	Feature that prompts the user to partake in a specific behavior through the use of a predetermined alert (see Figure 3)	Competence [cue to action]
Community forum	Feature that functions as a message board or chat room and allows likeminded individuals, whether patients with similar health conditions or their caregivers, the opportunity to share questions and experiences	Relatedness (social support, social norms); autonomy (acknowledging individual perspectives)
Social media	Feature that connects the user to Facebook, Twitter, or other social media platforms, thereby allowing the user to communicate progress with family, friends, colleagues, or others with ties to the user	Relatedness (social support, contextualization)
Addresses symptoms	Feature that addresses and assists in managing a disease that is associated with pain or other noticeable symptom(s)	Competence (educate, inform); autonomy (self-monitoring)
Tailored education	Feature that offers patient-specific education tailored to a person’s needs, interests and usage depending on his/her stage or progression of disease (eg, week of pregnancy)	Relatedness (personalization); competence (knowledge, skill development)
Tracker	Feature that allows for self-monitoring by recording information in order to modify personal attitudes or behaviors to achieve a predetermined goal or outcome (see Figure 4)	Autonomy (self-monitoring, self-regulation)
Cost	Identification of cost of the app (free, upfront payment, and/or in-app purchases)	N/A
Usability	Identification of satisfactory usability based on compliance with five interface design heuristics	Nielsen’s usability heuristics for user interface design

**Jakob Nielsen's five general principles for interaction design.**

Principle

1. Visibility of system status: app’s ability to keep users informed about what is going on and/or how they are progressing toward a goal.
2. User control and freedom: app provides the ability to easily control interactions, such as exit, save, go back, or edit.
3. Flexibility and efficiency of use: app provides the ability to accomplish intended tasks (eg, logging a meal or tracking blood pressure) quickly and efficiently.
4. Aesthetic and minimalist design: app is pleasant to look at and not overcrowded with irrelevant information.
5. Help users recognize, diagnose, and recover from errors: error messages within the app use plain language, simply state the problem, and outline steps to fixing it.

**Figure 1:**
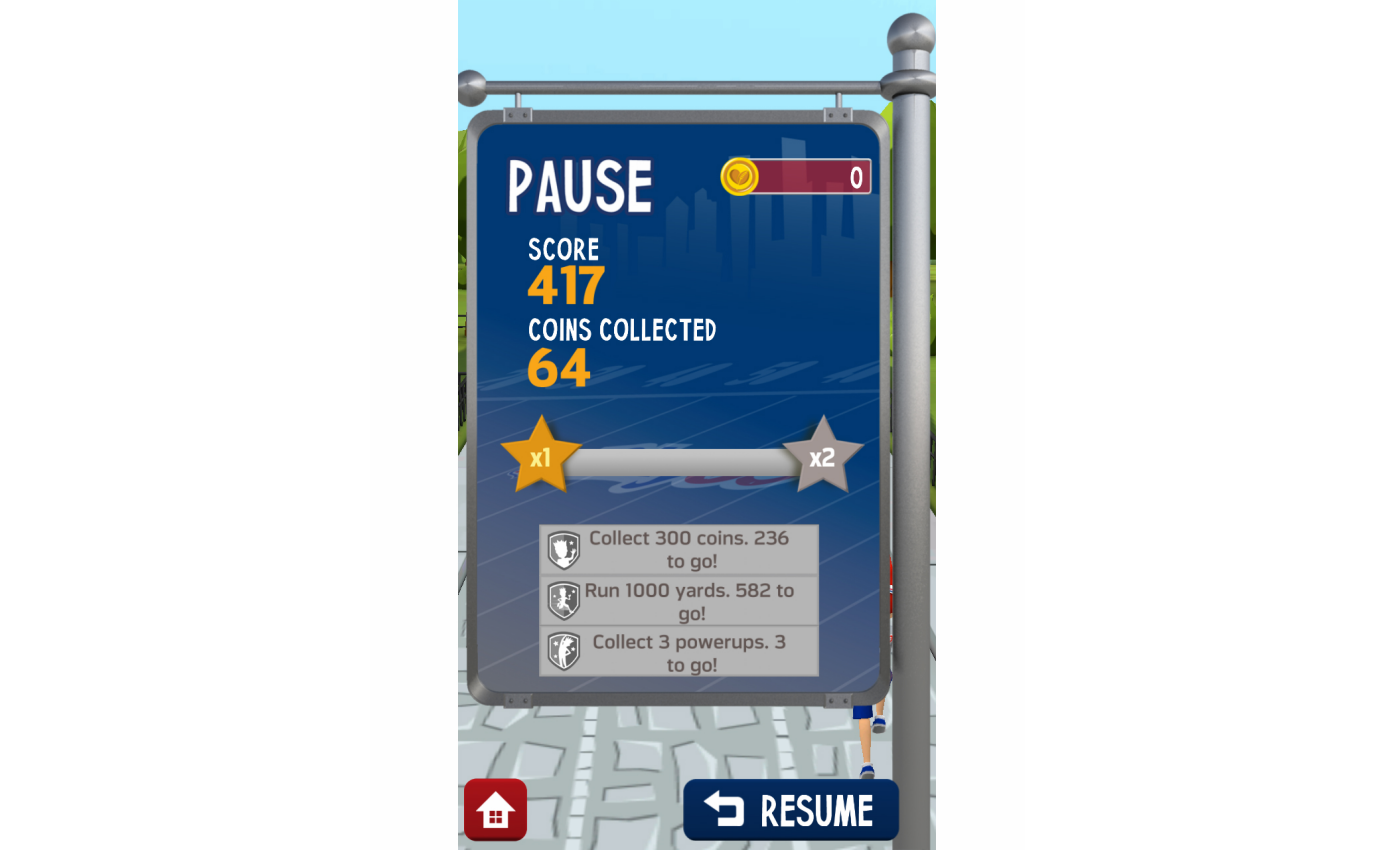
Gamification feature in the NFL PLAY 60 app.

**Figure 2:**
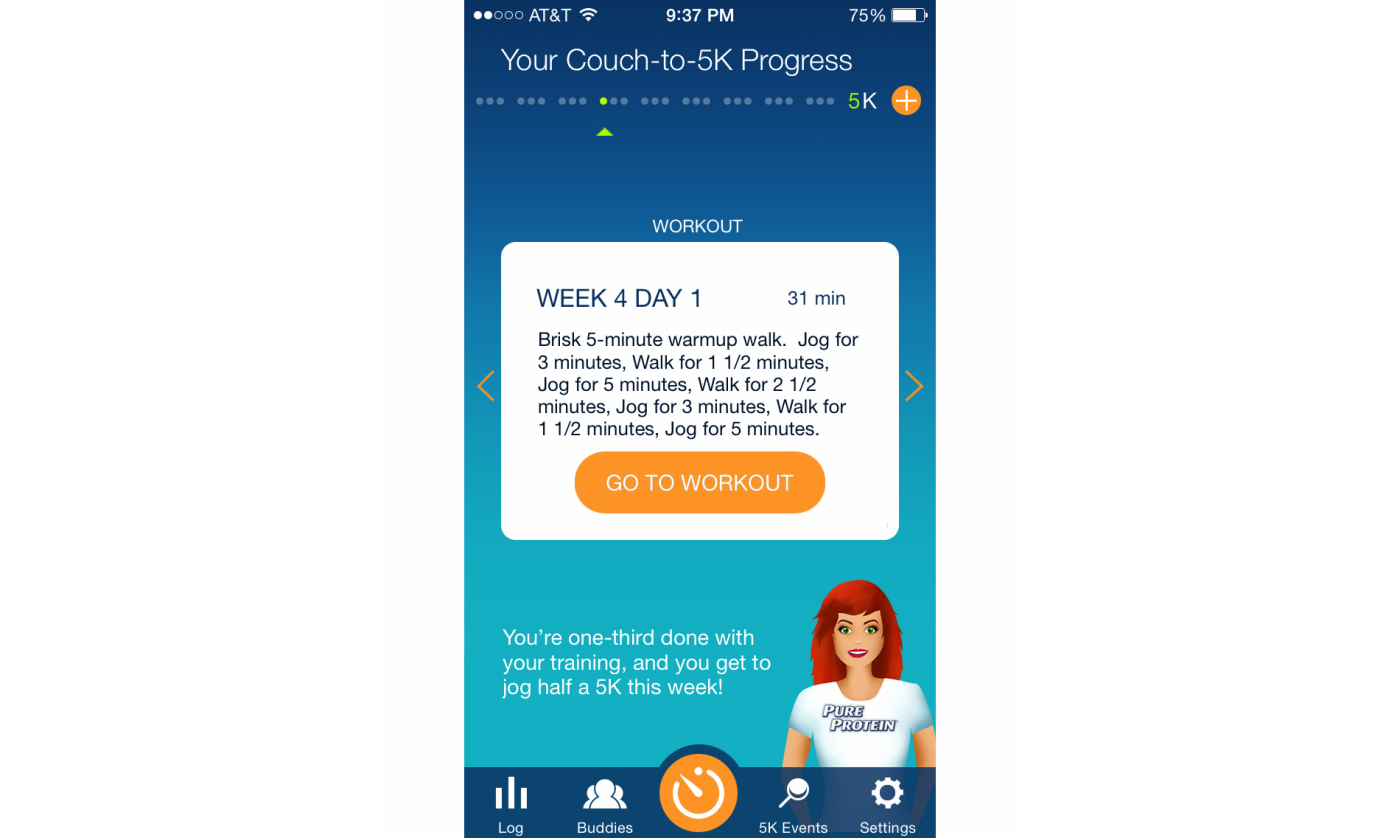
Plan or orders feature in the Couch-to-5K app.

**Figure 3:**
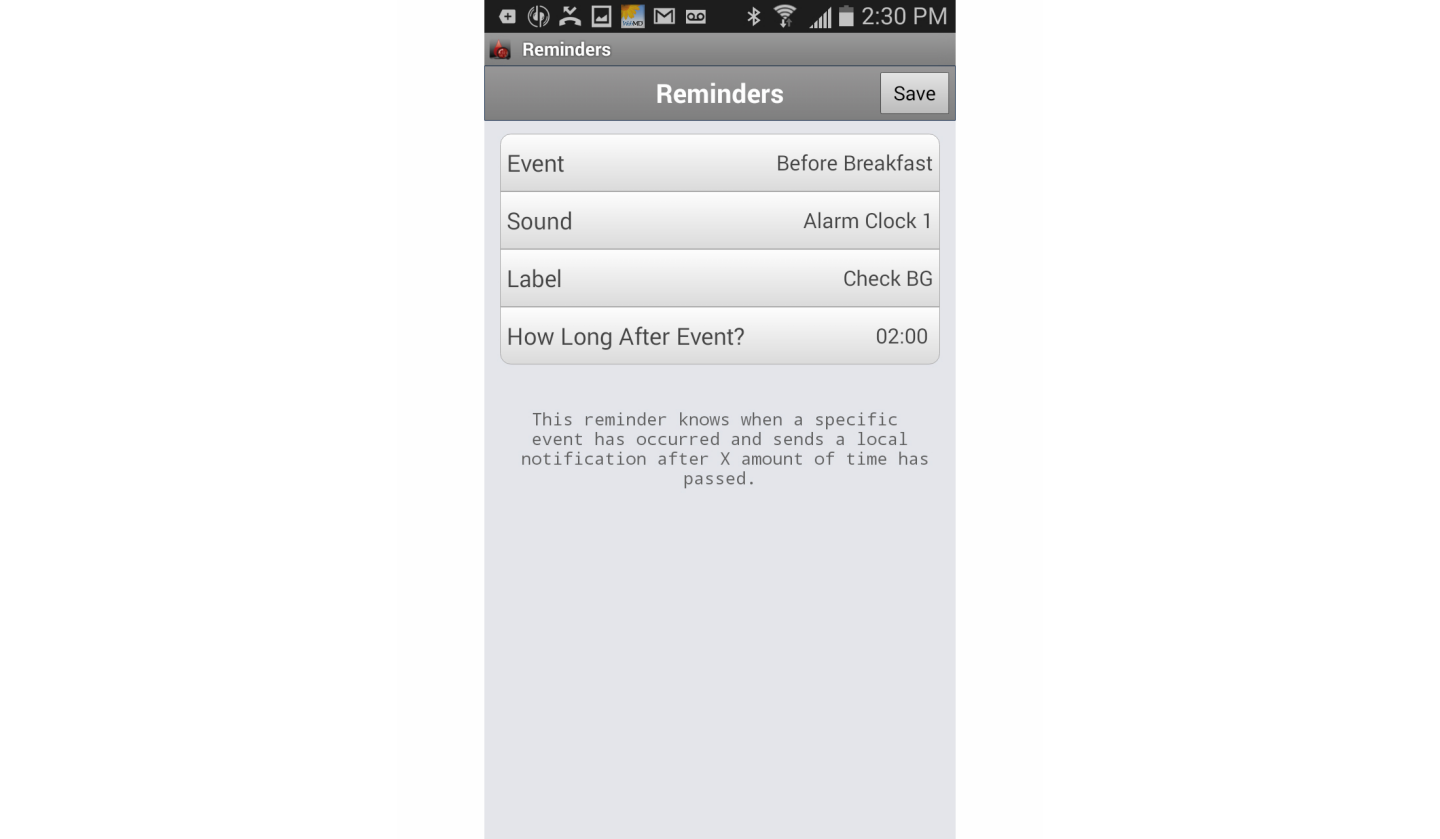
Reminder feature in the Glucose Buddy Diabetes Log app.

**Figure 4:**
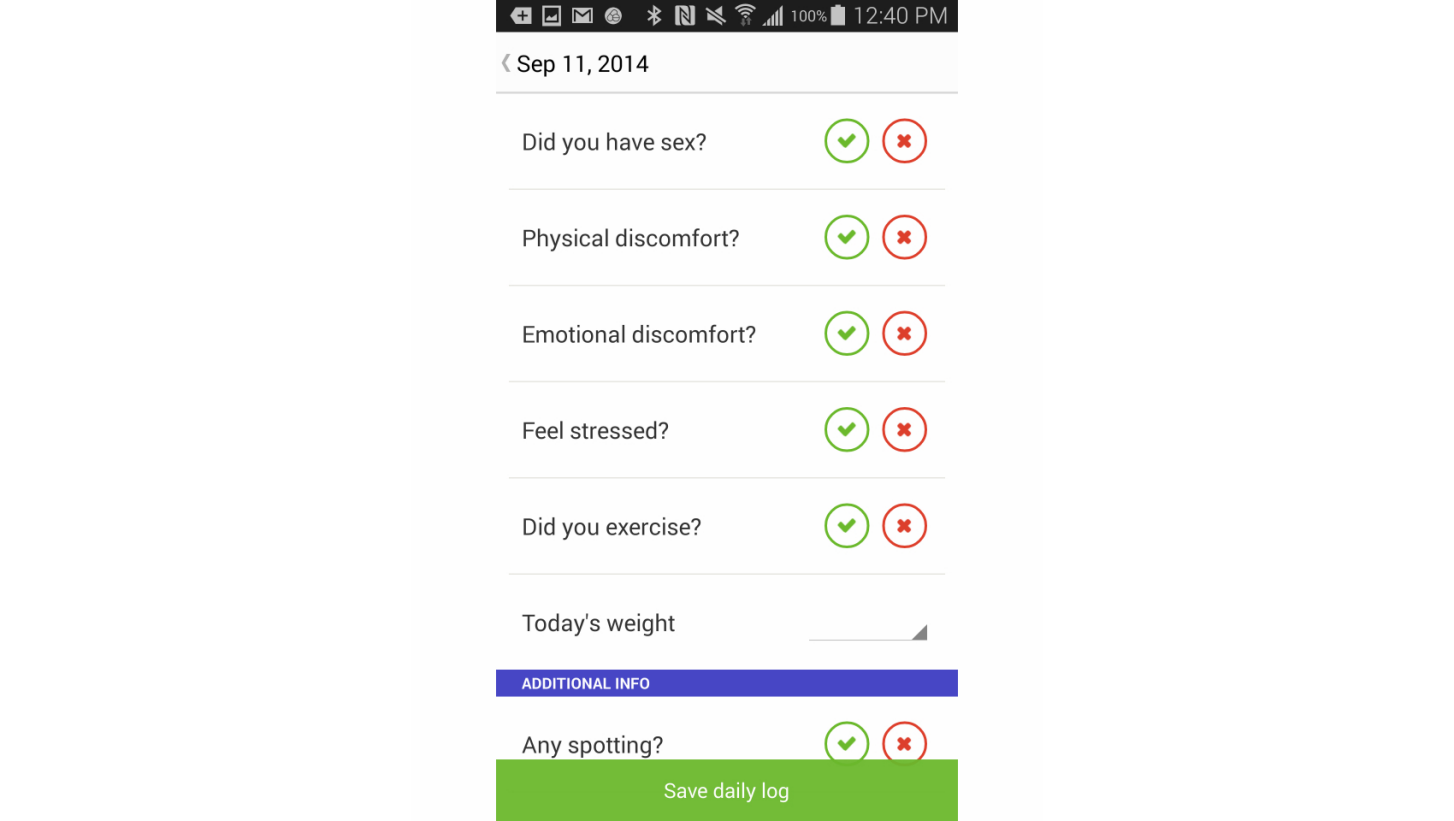
Tracking feature in the Glow Fertility & Ovulation app.

## Results

### General App Inclusion Criteria

**Figure 5:**
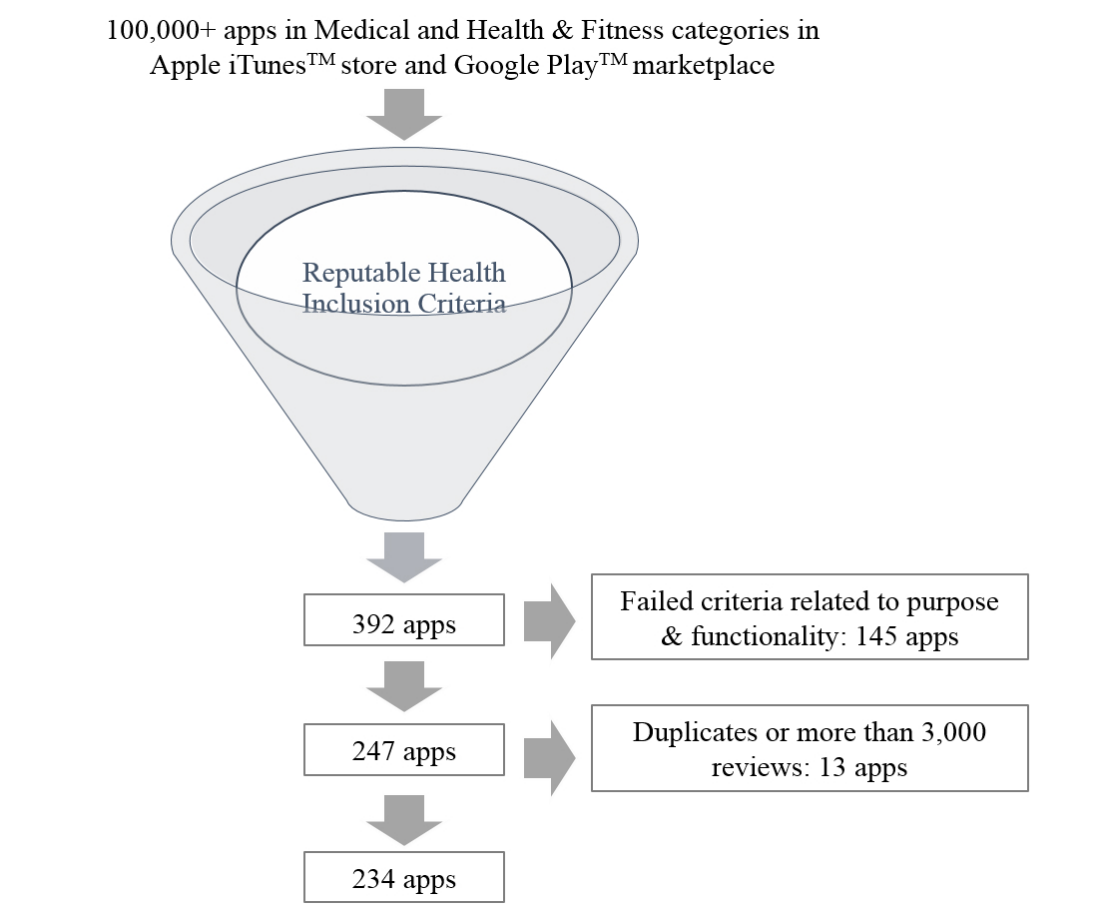
Flow Diagram of the app inclusion process.

Initially, 392 apps were identified by PubMed and mHealth Evidence, and the relational database provided by 42matters as having met the reputable health organization inclusion criteria. Figure 5 shows the elimination of apps at various stages throughout the initial app inclusion evaluation. Of the 392 apps, 145 were eliminated after not meeting the inclusion criteria related to purpose and functionality. Another 13 apps were then eliminated from consideration either because they were duplicates, or because of their unusually large number of reviews (ie, >3000 individual user ratings). Eliminating those with an unusually large number of reviews resulted in a slightly more explanatory model (*R* ^2^=.093 vs *R* ^2^=.090), with little change to the strength of the model (*F*=4.667 vs *F*=4.769) (Multimedia Appendix 1). The number of apps included in the final analysis was 234 (Multimedia Appendix 2). A breakdown of the total number and percentage of the 234 apps that met each of the inclusion criteria is displayed in Table 2. The reputable health organization inclusion criteria were each also treated as independent variables to confirm that they were not confounders, and once the analysis of the inclusion criteria showed no explanatory power (ie, <0.5%), those variables were eliminated from further analysis.

**Table 2:** Reputable health organization inclusion criterion totals (N=234).

Inclusion criteria	Apps, n (%)
Clinical trial/ research study	45 (19.2)
FDA-approved	8 (3.4)
Government-approved	16 (6.8)
US hospital system-approved	12 (5.1)
US academic medical institution-approved	21 (9.0)
Medical specialty society-approved	7 (3.0)
Non-profit health care organization-approved	39 (16.7)
Consumer organization with health focus-approved	68 (29.1)
US physician-approved	49 (20.9)
Third-party payer-approved	2 (0.9)
Pharmaceutical or medical technology company-approved	14 (6.0)

### Analysis of App Features and Characteristics

The 234 apps that remained were downloaded and manually assessed for the presence of certain features or characteristics. The findings of the app feature and characteristic assessment are shown in Table 3.

Simple linear regression analysis did not show that any single independent variable significantly impacted a user’s app rating. However, the features plan or orders, usability, cost, tracker, and gamification influenced the dependent variables to some degree (*R* ^2^ approximating ≥1% at a 95% significance level) (Table 4).

Multiple regression analysis was performed to investigate whether a combination of features, in particular, the aforementioned five features, could explain a user’s rating of an app. The same dependent and independent variables were used. The best model showed that 9.3% of the adjusted ratings (Y2) could be explained by plan or orders, usability, cost, tracker, and export of data (Tables 5 and 6).

The results of the correlation analysis are displayed in Table 7. Findings show a moderate positive correlation between the export of data and tracker features (.48), and a slight positive correlation between the tracker and usability features (.36). Any output <.2 or >-.2 was not considered significant.

**Table 3:** Assessment of the app features and characteristics (N=234).

App feature or characteristic	Apps, n (%)
Export of data	108 (46.2)
Gamification	27 (11.5)
General education	82 (35.0)
Plan or orders	41 (17.5)
Reminder	74 (31.6)
Community forum	46 (19.7)
Social media	61 (26.1)
Addresses symptoms	79 (33.8)
Tailored education	34 (14.5)
Tracker	170 (72.6)
Cost (free)	151 (64.5)
Usability	190 (81.2)

**Table 4:** *R* ^2^ and *P* value results for the simple linear regression analysis of individual app features at 95% significance level.

App feature	Y1^a^		Y2^b^		Y3^c^	
	*R* ^2^, %	*P* values	*R* ^2^, %	*P* values	*R* ^2^, %	*P* values
Plan or orders	4.2	<.001	4.3	<.001	4.0	<.001
Usability	1.3	.07	1.3	.07	1.7	.04
Cost	1.2	.08	1.0	.11	1.4	.06
Tracker	0.8	.18	0.8	.15	0.2	.48
Gamification	0.6	.21	0.7	.17	1.1	.09
Tailored education	0.4	.35	0.5	.28	0.5	.28
Addresses symptoms	0.3	.38	0.2	.48	0.3	.41
Reminder	0.3	.42	0.2	.45	0.3	.42
Community forum	0.2	.48	0.2	.47	0.4	.34
Export of data	0.2	.41	0.1	.51	0.3	.27
Social media	0.1	.65	0.1	.64	0.2	.53
General education	0.0	.98	0.0	.98	0.0	.75

^a^App store ratings

^b^Adjusted ratings

^c^Bayesian ratings

**Table 5:** Analysis of variance table with significance at the *P*<.05 level (N=234).

Source^a^	df^b^	*F*	MS^c^	*P*
Regression	5	4.667	(2.347)	*P*<.001
Residual	229		(.503)	

^a^Microsoft Excel

^b^Degrees of freedom

^c^Mean square

**Table 6:** Summary output for the multiple regression analysis to explain users’ app ratings (*R* ^2^= 0.093, 95% confidence level).

Source^a^	B^b^	SE B^c^	β^d^
Cost	.172	0.111	.103
Usability	.279^e^	0.130	.154^e^
Plan or Orders	.357^e^	0.127	.184^f^
Tracker	-.373^f^	0.125	-.226^f^
Export of Data	.226^e^	0.109	.151^e^

^a^Microsoft Excel

^b^Regression coefficient (beta)

^c^Standard Error of beta

^d^Standardized beta

^e^ *P*<.05

^f^ *P*<.01

**Table 7:** Correlation analysis of variables in the best model.

	Cost	Usability	Plan or orders	Tracker	Export of data
Cost	1				
Usability	-.06	1			
Plan or orders	.17	.02	1		
Tracker	.09	.36	-.10	1	
Export of data	.02	.22	-.19	.48	1

## Discussion

### Principal Findings

It was found that 9.3% of a user’s rating of an app can be explained by 5 app features or characteristics. Of these, plan or orders, export of data, usability, and cost contributed positively to a user’s rating, while the tracker feature impacted it negatively. Users value an app that is simple and intuitive to use, which aligns with Nielsen’s findings on usability [19]. Furthermore, users value tailored information and actionable insights regarding their condition and its management. This touches on both the autonomy and competence needs associated with SDT. Lastly, users want to be able to share their data with designated individuals, supporting the last basic psychological need of SDT, relatedness. In addition, the 4 app features that contributed positively to a user’s rating share one common theme: each provides a mechanism for care management that would appear to be less time-consuming and more efficient than current methods (Textbox 4).

**App features that contributed positively to a user's rating share a common theme.**

Feature

- Plan or orders
  - Users can save time by not having to investigate, decipher, and interpret the steps required to achieve a desired health goal, and in the process, appreciate immediate access to viewing their progress.
- Export of data
  - Users understand the value of sharing their progress (and setbacks) with their health care provider, and appreciate the time saved by not needing to input data into an email or having a member of the health care provider’s staff copy the results into a health record.
- Usability
  - Users value the layout of an app that is efficient, intuitive, and allows for easy input of information.
- Cost
  - Users rate paid apps consistently higher than free apps, presumably because paid apps are usually void of advertisements, (ie, the main revenue source for most free apps), which can lead to a more efficient experience.

Although the fifth feature, tracker, returned a negative coefficient, further analysis revealed that the tracker feature is positively correlated with the export of data and the usability features. The moderate positive correlation between the tracker and export of data features (.48) may indicate that the ability to track progress isn’t valuable to the user without the ability to transfer the data collected. Since a large majority of apps are able to both track and export data, an app that doesn't have both components is likely outdated or lacks sophistication, and thus may not be rated highly. It was determined that of the 234 apps studied as part of this research, 180 (76.9%, 180/234) contained a tracker feature. Of those, 62 (34.4%, 62/180) did not provide a method to export the data to a website, email, or electronic health record.

A moderate positive correlation between the tracker and the usability features (.36) strengthens the argument that the process of entering information into the tracker function of an app, as well as the value of the output display of the data collected, may be of great importance when a user assesses the tracker feature. Any further research to better understand the relationship between the overall user experience and tracking should begin by focusing on the differences between active tracking through the manual input of information and passive tracking where data is collected through sensors or devices.

Interestingly, popular and well-studied features such as gamification and the ability to connect to social media did not appear to influence a user’s rating of apps in this analysis. These results were unexpected, particularly since social media and gaming apps are consistently the most downloaded and used apps on mobile phones [20]. The findings do not conclude that the aforementioned features are not valuable in engaging a patient, changing behavior, or improving outcomes, but solely that they do not seem to factor in the rating of the apps reviewed. However, these features seem poised to play a pivotal role in the future of digital health.

### Limitations

#### Overview

Although the list of app features and characteristics compiled in order to explain user ratings was fairly exhaustive, this analysis does not account for >90% of an app rating. Potential reasons for this discrepancy are discussed in the following sections.

#### One-Size Fits All

Similar to other solutions in health care, apps are not a one-size-fits-all answer. Different users will value different features, layouts, and approaches.

#### Combining Apps

This analysis intentionally combined and analyzed apps from the medical, health, and fitness categories of the app stores. It is likely that users may rate a feature as valuable for one category of apps that may be irrelevant or detrimental to another, thereby negating its value in the overall analysis. For example, a reminder feature is essential for medication trackers, but it may be counterproductive in a smoking cessation app. Additional research is needed to focus on one category in the app store, specific diseases [8,21], or the desired behavior change; eventually, it may be determined that different evaluation criteria are needed for different types of apps.

#### Quality

This investigation focused on the presence or absence of most of the app features without evaluating the quality of the feature. A brief, informal examination of app store reviews for 10.3% (24/234) of the apps analyzed in this study (chosen randomly) showed that users often expressed displeasure with features of poor quality. The mere presence of a feature does not assure its value to the user; future app feature research should likely include a qualitative component, and overall user experience should be taken into account.

#### Rating Systems

The process of rating an app in the Apple iTunes store is more complex than in the Google Play marketplace [22], which may explain why the Google Play marketplace had, on average, a higher number of user ratings for the same app. An attempt to address this issue was made by omitting apps with <25 user ratings in the app store, but it is possible that the differences in rating processes may have impacted the results.

#### Patient-Consumers

The only apps included in this analysis were those intended for use by patient-consumers. This determination was made by a single reviewer, who downloaded each app, explored the features and functions, and subjectively determined the intended audience. Having had multiple reviewers participate in this assessment would have helped the process be more objective.

#### Usability

Although rooted in Nielsen’s usability heuristics, some of the usability principles assessed are subjective by nature (eg, aesthetic and minimalist design). Having multiple reviewers participate in the assessment would have helped the process be more objective.

#### Number of Ratings

Reports have raised some concerns as to the legitimacy of the quantity of ratings and reviews for some mobile apps, which may confound results. Although precautions were taken by omitting apps from the analysis that contained an unusually large number of ratings (ie, >3000 individual user ratings) in order to reduce the potential of confounding, some illegitimate app ratings may have gone undetected and altered the findings.

### Future Research

Gamification deserves more attention and study, particularly as a method to engage adults with chronic conditions. One-third of adults between 30-49 years old have at least one chronic condition compared with 60% of adults aged 50-64 years [23], with the majority being female [24]. This would indicate that mHealth app features that assist in disease management should be tailored to an older demographic and slightly more to women. Although some may postulate that gamification would not appeal to older generations of adults, McKinsey's Global iConsumer research found that approximately 50% of casual gamers are between 35-64 years of age, 54% are female, and the majority will stick to the same casual games for >6 months [25]. Lastly, at the time of the study, the app developers in the examined dataset did not offer incentives or rewards for reaching milestones. As such, further investigation into gamification coupled with a rewards and/or incentive program is warranted.

Similarly, social media seems ideally suited for mHealth engagement, even though the ability to access social media through an app did not impact the app’s rating. Research by the Pew Internet Project indicated that as of January 2014, nearly 75% of those accessing the Internet also use social media [26], and a 2012 PwCHealth study showed that nearly one-third of those surveyed would be interested in having their social media conversations monitored if it would help them improve their health or better coordinate care [27]. Even at its lowest levels of adoption, approximately 65% of individuals between the ages of 50-65 use some form of social media (compared to nearly 90% in younger demographics) [26]. Therefore, age does not seem to be a limiting factor for integrating social media into health.

However, social media’s role in this analysis may not be representative of its true value. Until recently, third-party private health insurance plans could deny coverage to patients with pre-existing conditions or insure them at significantly higher premiums. Sharing personal health information publicly carried financial concerns related to insurance status, potentially explaining why people would be more hesitant to share their health information in the same way that they share other personal information. With the implementation of PPACA and the elimination of pre-existing condition exclusions, social media may yet play a much larger role in transforming and managing care. Undoubtedly, deeper exploration is necessary to gain a better understanding of the roles that social media and gamification can play in unlocking the true potential of mHealth solutions for better health management.

Clearly, additional research must be conducted to expand the scope of mHealth apps reviewed and better understand what aspects and features are most valuable to its users. Deeper investigations and varied approaches are necessary to determine the roles of future versions of gamification, incentive programs, social media, and trackers within defined app categories in the app store, specific diseases, and desired behavior changes. Furthermore, similar analyses examining apps within only one condition or wellness category (eg, asthma management or nutrition) is necessary. This would allow for the investigation of more specific app features and has the potential to yield stronger findings. Lastly, a deeper dive into the impact of usability on user ratings is warranted. Since this study chose to focus largely on behavioral science features, it may also be useful to better understand the relative impact of each of Nielsen’s 10 usability principles on a user’s rating of health apps.

### Implications and Recommendations

The field must keep working to move toward developing more sophisticated and better integrated digital tools in order to gain overall user acceptance, sustained engagement, and ultimately, clinical value and behavior change. As the digital health industry evolves, users will be able to collect more data and achieve better results while having to actively coordinate, input, and transmit less activity. Based on ratings of apps associated with reputable health organizations, users find some value in apps and features that save them time and effort, but additional research is critical in order to maximize digital health’s potential while advancing the triple aim of health care to improve access and increase patient satisfaction while lowering overall costs.

## Multimedia Appendix 1

Side-by-side comparison of multiple regression models to explain users’ app ratings.

## Multimedia Appendix 2

The 234 mobile health (mHealth) apps that met the inclusion criteria and guidelines.

## Abbreviations

mHealth: Mobile health
PPACA: Patient Protection and Affordable Care Act
SDT: Self-Determination Theory
