# Supplementary material for: Valuable Features in Mobile Health Apps for Patients and Consumers: Content Analysis of Apps and User Ratings
Source: JMIR Mhealth Uhealth. 2015 May 13;3(2):e40. doi: 10.2196/mhealth.4283 (PMC4446515; doi:10.2196/mhealth.4283)
Supplement: Supplementary file 1 [file mhealth_v3i2e40_app1.pdf]

Multimedia Appendix 1: Side-by-Side Comparison of Multiple Regression Models to Explain Users' App Ratings

Source: Microsoft Excel	Final Model / n=234 apps			Model / n=247 apps		
	B	SE B	β	B	SE B	β
Cost	0.172	0.111	.103	0.155	0.108	.091
Usability	0.279[a]	0.130	.154[a]	0.307[a]	0.126	.162[a]
Plan or Orders	0.357[a]	0.127	.184[b]	0.360[b]	0.121	.189[b]
Tracker	-0.373[b]	0.125	-.226[b]	-0.335[b]	0.120	-.205[b]
Export of Data	0.226[a]	0.109	.151[a]	0.329	0.184	.127
	R^2= 0.093			R^2= 0.090		
	F= 4.667			F= 4.769		

Notes: 95% Confidence Level.

[a]*P < .05.* [b]*P < .01*.
