# Supplementary material for: Valuable Features in Mobile Health Apps for Patients and Consumers: Content Analysis of Apps and User Ratings
Source: JMIR Mhealth Uhealth. 2015 May 13;3(2):e40. doi: 10.2196/mhealth.4283 (PMC4446515; doi:10.2196/mhealth.4283)
Supplement: Supplementary file 2 [file mhealth_v3i2e40_app2.pdf]

Multimedia Appendix 2: The 234 Mobile Health Applications That Met Inclusion Criteria and Guidelines

1.
2. All-in Fitness: 1200 Exercises, Workouts, Calorie Counter, BMI calculator by Sport.com, iOS by Plus Sports
3. ARGUS - Motion and Fitness Tracker by Azumio, iOS by Azumio Inc.
4. ASCVD Risk Estimator, Android by American College of Cardiology Foundation
5. AsthmaMD, iOS by Mobile Breeze
6. Attain Fertility® Planner, iOS by IntegraMed America
7. Autism Learning Games: Camp Discovery (iPad), iOS/Android by Center for Autism and Related Disorders
8. Baby • Sprout, iOS by Med ART Studios
9. Baby 411 by Dr. Ari Brown, iOS by Baby 411 / Windsor Peak
10. Baby Bundle, iOS by Nicab Inc
11. Baby Sleep Tracker • Sprout, iOS by Med ART Studios
12. Baby Tracker from WhatToExpect.com, iOS by Everyday Health, Inc.
13. BabyTime Contraction Timer, iOS by Vanderbilt University
14. bant - A diabetes app for the ePatient, iOS by University Health Network
15. Best Android Symptom Checker, Android by Senstore - Powered by Harvard Medical School
16. Blood Sugar Diabetes Control, iOS by G.P. Imports, Inc.
17. Blood Sugar Tracker by healthycloud.com, iOS by Azumio Inc.
18. BlueLoop, Android by MyCareConnect
19. Body-for-LIFE, Android by Abbott
20. Breast Cancer Diagnosis Guide, iOS by Breastcancer.org
21. Breast Cancer: Beyond the Shock, iOS by National Breast Cancer Foundation
22. Breathe2Relax, iOS/Android by The National Center for Telehealth and Technology
23. Breathing Zone - Relaxing Breathing Exercises, iOS by Breathing Zone
24. Calcium Pro, iOS by Calcium Science
25. Calorie Counter, Android by Everyday Health
26. Calorie Counter, Android by CalorieCount.com
27. Calorie Counter & Diet Tracker, Android by SparkPeople
28. Calorie Counter by FatSecret, iOS/Android by FatSecret
29. Calorie Counter PRO by MyNetDiary, iOS/Android by MyNetDiary Inc.
30. Calorie Counter, Dining Out, Food, and Exercise Tracker, iOS by Everyday Health, Inc.
31. Cancer.Net Mobile, iOS/Android by ASCO
32. Carb Counting with Lenny, Android by Medtronic, Inc.
33. Cardiio - Heart Rate Monitor, 7 Minute Workout, Calorie Burn Tracker, iOS by Cardiio, Inc.
34. CaringBridge, Android by CaringBridge.org
35. CaringBridge, iOS by CaringBridge.org
36. Carolinas Health System, iOS/Android by Carolinas HealthCare System
37. CDC, iOS by Centers For Disease Control and Prevention
38. ChildrensMD, iOS by Children's Hospital Colorado
39. Cigarettes, iOS by Thomas Kiesl
40. Cigarettes LITE, iOS by Thomas Kiesl
41. Cleveland Clinic Stress Meditations, iOS by Cleveland Clinic Wellness Enterprise
42. Cleveland Clinic Today, iOS by Cleveland Clinic Innovations
43. Cody - Fitness Community, Exercise Journal, Workout Tracker, iOS by Cody Inc
44. CogniFit Brain Fitness, iOS by CogniFit
45. CogniFit Brain Fitness for iPad, iOS by CogniFit
46. Couch-to-5K, iOS/Android by The Active Network, Inc.
47. CPR QuickGuide, Android by todohw
48. CPR•Choking, Android by LearnCPR
49. CVS Pharmacy, iOS/Android by CVS Pharmacy
50. Depression Test, Android by Japps Medical
51. Dermatology A-Z, Android by American Academy of Dermatology

52. Diabetes App - blood sugar control, glucose tracker and carb counter, iOS by BHI Technologies, Inc.

53. Diabetes App Lite - blood sugar control, glucose tracker and carb counter, iOS by BHI Technologies, Inc.

54. Diabetes Companion by MySugr, iOS by mySugr GmbH

55. Diabetes Companion by mySugr, Android by mySugr GmbH

56. Diabetes Diary, iOS by fridayforward

57. Diabetes in Check, iOS by Everyday Health, Inc.

58. Diabetes in Pregnancy - Gestational Diabetes Logbook, Diabetes Manager, Pregnancy Diabetes Tracker, iOS by Coheso, Inc.

59. Diabetes Log, iOS by Distal Thoughts

60. Diabetes Pal App: Logbook Manager for Blood Glucose, A1c, Nutrition, Medication, Weight, Blood Pressure Analysis + Withings and BodyMedia, iOS/Android by Telcare, Inc.

61. Diabetes Pilot, iOS by Digital Altitudes, LLC

62. Diabetes Tracker with Blood Glucose/Carb Log by MyNetDiary, iOS by MyNetDiary Inc.

63. Diamedic, iOS by Nicholas Martin

64. Diet & Food Tracker - SparkPeople, iOS by SparkPeople, Inc.

65. DoseCast, iOS by Montuno Software, LLC

66. EarMachine, iOS by Ear Machine LLC

67. Emergency First Aid & Treatment Guide, iOS by phoneflips

68. #N/A

69. Every Body Walk!, iOS/Android by Kaiser Permanente

70. Everyday Health for iPad, iOS/Android by Everyday Health, Inc.

71. Everyday Health: Health News and Medical Information, iOS/Android by Everyday Health, Inc.

72. ExpectingBaby by Enfamil® Pregnancy Journal, iOS/Android by Mead Johnson & Company, LLC

73. FibroCite for Fibromyalgia, Android by OSInt health

74. Fig- your personal wellness guide for body and soul, iOS by Fig.com, Inc.

75. FitBit, iOS by Fitbit, Inc.

76. FitBit, Android by Fitbit, Inc.

77. Fitness Buddy: 1700 Exercises Workout Journal, iOS/Android by Azumio Inc.

78. Food and Fluid Daily Diary, iOS by Gilead Sciences, Inc.

79. Foods to Avoid When Pregnant, iOS by LitCharts

80. Fooducate - Healthy Weight Loss, Diet Tracker & Food Scanner, iOS/Android by Fooducate, Ltd.

81. Foot Decide - Point of Care Patient Education for Healthcare Professionals by Orca Health, iOS by Orca Health, Inc.

82. GenieMD, iOS by GenieMD, LLC

83. Gerber Pregnancy Calendar, iOS by Gerber Products Company

84. GI Buddy, iOS by Crohn’s & Colitis Foundation of America (CCFA)

85. GI Monitor, iOS by Medivo Inc

86. GI Monitor, Android by Medivo

87. Glooko, iOS by Glooko, Inc.

88. Glow - Period, fertility, ovulation tracker, iOS by Glow

89. Glow Fertility & Ovulation, Android by Glow Inc

90. Glucose Buddy - Diabetes Logbook Manager w/syncing, Blood Pressure, Weight Tracking, iOS by Azumio Inc.

91. Glucose Buddy : Diabetes Log, Android by Azumio, Inc.

92. Glucose Buddy Pro : Diabetes Managing Logbook w/ Blood Pressure & Weight Tracking, iOS by Azumio Inc.

93. Glucose Companion, iOS by Maxwell Software

94. Glucose-Charter, iOS by e-Agent

95. GoMeals, iOS/Android by sanofi-aventis U.S. LLC.

96. GymGoal 2, iOS by Maryna Kolokolkina

97. GymGoal Free, iOS by Maryna Kolokolkina

98. Hand Decide - Point of Care Patient Education for Healthcare Professionals by Orca Health, iOS by ORCA MD

99. Hands-Only CPR, iOS by Jive Media LLC

100. Healthy Children, Android by American Academy of Pediatrics

101. Healthy Habits ™, iOS by 2Morrow Mobile

102. Healthy Heart 2, iOS by Ringful LLC

103. HealthyOut Healthy Meal Finder, Android by HealthyOut

104. Heart Health Mobile, iOS by Marshfield Clinic

105. Heart Pal Free - Blood Pressure Tracker, iOS by Deltaworks
106. HeartWise Blood Pressure Tracker, iOS by SwEng L.L.C.
107. higi - Your Score for Life, iOS by higi llc
108. I AM LOVE - Kids' Yoga Journey, iOS by Gramercy Consultants
109. I'm Expecting Pregnancy App and Baby Guide - Calendar, Symptoms Tracker, Weekly Videos, and Baby Bump Photo Journal for Pregnant Moms until your Due Date, iOS by MedHelp
110. IAmGeneress, iOS by Watson Pharmaceuticals
111. iBGStar® Diabetes Manager, iOS by sanofi-aventis U.S. LLC.
112. iBP Blood Pressure, iOS/Android by Leading Edge Apps LLC
113. iCPR Full, iOS by D-Sign
114. iHeadache - Free Headache & Migraine Diary App, iOS by BetterQOL.com
115. iHeadache - Headache & Migraine Diary , iOS by BetterQOL.com
116. iHealth BPM, iOS by ANDON HEALTH Co., LTD
117. iHealth MyVitals, iOS by iHealth Lab Inc.
118. iHealth MyVitals, Android by iHealth Lab Inc.
119. iHeart Touch, iOS by Nucleus Medical Media
120. iPeriod Period Tracker Ultimate / Menstrual Calendar, iOS by Winkpass Creations, Inc.
121. iQuit - Stop Smoking Counter, iOS by Vidal de Wit
122. Is My Food Safe?, iOS by Academy of Nutrition and Dietetics
123. iTriage - Health, Doctor, Symptoms and Healthcare search, iOS/Android by Healthagen LLC
124. Jillian Michaels Slim-Down, Android by Everyday Health
125. Jillian Michaels Slim-Down: Weight Loss, Diet, & Exercise Solution, iOS by Everyday Health, Inc.
126. JOHNSON'S® Baby BEDTIME™ Sleep, iOS by Johnson & Johnson Consumer Companies, Inc.
127. Kid Care - from St. Louis Children's Hospital, iOS/Android by 3CN Self Care
128. Kids' Wellness Tracker, iOS/Android by McNeil-PPC, Inc
129. KidsDoc - from the American Academy of Pediatrics, iOS by Self Care Decisions, LLC
130. Kindara Fertility Tracker, Ovulation Calculator & Basal Body Temperature Chart - to Help You Get Pregnant Naturally with the Fertility Awareness Method of Natural Family Planning, iOS by Kindara, Inc.
131. Knee Decide - Point of Care Patient Education for Healthcare Professionals by Orca Health, iOS by Orca Health, Inc.
132. Lets Move It, iOS by Cleveland Clinic Wellness Enterprise
133. LIVESTRONG Calorie Tracker, Android by LIVESTRONG
134. LIVESTRONG MyQuit Coach - Dare to Quit Smoking, iOS by Demand Media, Inc.
135. LIVESTRONG.COM - Calorie Tracker LITE - Your Free Diet and Fitness Calorie Counter for Better Health, iOS by Demand Media, Inc.
136. Lose It!, Android by FitNow, Inc.
137. Low Carb Diet Assistant, iOS by nanobitsoftware.com
138. Luna Tracker™, Android by Sunovion Pharmaceuticals Inc
139. Mango Health - Medication Manager, Pill Reminder, Drug Interactions, iOS by Mango Health
140. MapMyWalk GPS Walking, Android by MapMyFitness, Inc.
141. Mayo Clinic Meditation, iOS by Mayo Clinic Health Solutions
142. MedCoach Medication Reminder, iOS/Android by GreatCall, Inc.
143. MediSafe Medication and Pill Reminder - top ranked drug management app for individuals and families adherence and compliance, iOS/Android by MediSafe Project
144. Meds Agenda, iOS by Intersog
145. MedSimple, iOS/Android by My Diabetes Home, LLC
146. MedWatcher for drugs, vaccines and medical devices, iOS by John Brownstein
147. Moody Me - Mood Tracker and Diary, iOS by MedHelp
148. Moves, Android by ProtoGeo
149. Moves , iOS by ProtoGeo
150. My Diabetes, Android by Rossen Varbanov
151. My Healthy Habits, iOS by Indiana University Health
152. My Meal Mate, Android by X-Lab Ltd

153. My Medications, iOS by American Medical Association
154. My Migraine Triggers, iOS/Android by Novartis Consumer Health
155. myBG Lite, iOS by Beachdog Enterprises
156. MyDS, iOS by NIH Office of Dietary Supplements
157. MyEpilepsyDiary, iOS/Android by Irody Inc.
158. myFitnessCompanion, Android by myFitnessCompanion
159. MyMedSchedule, Android by MyMedSchedule
160. myPill® Reminder, Android by Bouqt
161. myPill® Reminder - Pill, Ring or Patch contraceptive, Period tracker, Discreet message, Calendar, Custom alarm, all birth control types!, iOS by Bouqt.com Ltd
162. Nexercise - motivation to lose weight, to finally meet your weight loss & health goals, iOS/Android by Nexercise
163. NFL PLAY 60, iOS/Android by American Heart Association
164. Nike Training Club, iOS by Nike, Inc.
165. Noom Weight Loss, iOS by Noom, Inc.
166. Nutrition Menu - Calorie, Exercise, Weight & Water Tracking, iOS by Shroomies
167. OnTrack Diabetes, Android by Medivo
168. Pain Care, iOS/Android by Ringful LLC
169. Pampers Hello Baby Pregnancy Calendar, iOS by P&G Productions
170. Parkinson's Central, iOS by National Parkinson Foundation
171. Patient, iOS/Android by Mayo Clinic
172. Period and Fertility Tracker from WhatToExpect.com, iOS by Everyday Health, Inc.
173. PhotoCalorie, iOS by PhotoCalorie
174. PhysioAdvisor Exercises, Android by PhysioAdvisor
175. Pillboxie, iOS by Jared Sinclair
176. Pocket First Aid & CPR from the American Heart Association, iOS/Android by Jive Media LLC
177. Pocket Yoga, iOS/Android by Rainfrog, LLC
178. Power 20 Fitness Trainer FREE, Android by Power 20
179. Power 20 Fitness Trainer Pro 20-Minute Daily Workout, iOS by Power 20
180. Pregnancy • Sprout, iOS by Med ART Studios
181. Pregnancy • Sprout • Lite, iOS by Med ART Studios
182. Pregnancy Due Date & Fertility Calculator, Tools and Baby Kick App, iOS by BabymedLLC
183. Pregnancy Tracker, Android by Everyday Health
184. Proactive Sleep Alarm Clock, iOS by Proactive Life LLC
185. PTSD Coach, iOS/Android by US Department of Veterans Affairs (VA)
186. Quit Smoking - Cold Turkey, iOS by Pinch Swipe Tap Pty. Ltd.
187. Quit Smoking - Cold Turkey (Lite Version), iOS by Pinch Swipe Tap Pty. Ltd.
188. Quit Smoking Health Counter HD, Android by MobiGeni.com
189. Quit Smoking Helper, iOS by Tae-han Kim
190. Quit Smoking Now with Max Kirsten, iOS by Craig Ray
191. Quit Smoking with Andrew Johnson, iOS/Android by Michael Schneider
192. Quitter, iOS by Paze
193. Resuscitate! CPR AED & Choking, iOS by Stone Meadow Development LLC
194. Run 10k - interval training coach + stretch program, iOS by Felt Tip Inc.
195. RunKeeper - GPS Track Running Walking Cycling, iOS/Android by FitnessKeeper, Inc.
196. Short Sequence: Kids' Yoga Journey Lite, iOS by Gramercy Consultants
197. SiDiary Diabetes Management, Android by SINOVO Ltd. & Co. KG
198. Similac Baby Journal, iOS by Abbott
199. Simply Being - Guided Meditation for Relaxation and Presence, iOS/Android by Meditation Oasis
200. Simply Sayin', iOS by MediaKube, LLC
201. Sleep Time - Alarm Clock and Sleep Cycle Analysis with Soundscapes, iOS by Azumio Inc.
202. Sleep Tracker TYLENOL® PM, iOS by TYLENOLÂ®
203. SleepBot - Sleep Cycle Alarm, Android by SleepBot
204. SleepBot - Smart Cycle Alarm with Motion & Sound Tracker, iOS by SleepBot
205. Soundamp R, iOS by Ginger Labs
206. South Beach Diet, Android by Everyday Health

207. South Beach Diet ®, iOS by Everyday Health, Inc.
208. Spot a Stroke F.A.S.T., Android by American Heart Association, Inc
209. Stop Smoking Cigarettes Now Quit Smokes Forever Tracker, Counter, & No Smoker Cigarette Quitter Coach App, iOS by Ellisapps Inc.
210. Stress Check by Azumio, Android by Azumio Inc.
211. Stress Check Pro by Azumio, iOS by Azumio Inc.
212. Stress Free with Andrew Johnson, iOS by Michael Schneider
213. Stress Free with Deepak Chopra, iOS by Signal Patterns
214. T2 Mood Tracker, iOS/Android by The National Center for Telehealth and Technology
215. Tactical Breather, Android by T2
216. Tap & Track -Calorie Counter (Diets & Exercises), iOS by nanobitsoftware.com
217. The EX Plan, iOS by American Legacy Foundation
218. The Snack App, iOS by Everyday Health, Inc.
219. TRACK + REACT, iOS by Arthritis Foundation
220. TRACK + REACT, Android by Arthritis Foundation
221. Track3 - Diabetes Planner, Diabetes Glucose Logbook, Diabetes Tracker, Carb Counter, iOS/Android by Coheso, Inc.
222. Tummy Trends: Constipation and Irritable Bowel Syndrome Tracker, iOS by Takeda Pharmaceuticals
223. Walk with Map My Walk - GPS Walking, Jogging, Running, Workout Tracking for Diet Weight Loss, iOS by MapMyFitness
224. WaveSense Diabetes Manager, iOS by AgaMatrix
225. WebMD Allergy, iOS by WebMD
226. WebMD Baby, iOS/Android by WebMD
227. WebMD Pain Coach, iOS/Android by WebMD
228. WebMD Pregnancy , iOS by WebMD
229. Wellness Tip of the Day, iOS by Cleveland Clinic Wellness Enterprise
230. WhatsMyM3, iOS by M-3 Information, LLC
231. WiThings Health Mate, iOS/Android by WiThings, S.A.S.
232. Workout Trainer, iOS by Skimble
233. Wrist Repair, Android by Meditech Communications, Inc.
234. Zyrtec AllergyCast, iOS by McNeil-PPC, Inc
235. ZYRTEC® AllergyCast, Android by McNeil-PPC
